# Supplementary material for: Fermentation technology as a driver of human brain expansion
Source: Commun Biol. 2023 Nov 23;6:1190. doi: 10.1038/s42003-023-05517-3 (PMC10667226; doi:10.1038/s42003-023-05517-3)
Supplement: Supplementary file 2 — Supplementary Information [file 42003_2023_5517_MOESM2_ESM.pdf]

Food Name	Food Type	Fermentation Type	Origin	Incubation Time	Medium	Microflora	Source
kimchi	vegetable (many kinds)	acid	Asia	1 month	underground pots	Lactic acid bacteria (LAB)	Lee et al., 1986
sauerkraut	vegetable (leaves)	acid	Europe	weeks to months	salted and packed in jars with brine	Enterobactericiae, LAB, yeasts	Fleming et al., 1988; Stamer et al., 1971
puerh (aged black tea)	vegetable (leaves)	acid	Asia	weeks to months	dried and compressed	Aspergillus, Penicillium, Rhizopus, and Saccharomyces spp.	Wang et al., 2011; Lv et al., 2013
grape leaves	vegetable (leaves)	acid	Europe/Middle East	weeks to months	soaked	LAB	Basoglu et al., 1996; Kosar et al., 2007
sinki	vegetable (tuber)	acid	India, Nepal	weeks	dried and packed in jars	LAB	Tamang and Sarkar, 1993
gari	vegetable (tuber)	acid	West Africa	several days	grated and placed in knit bags in a trench	LAB, yeasts, fungi	Westby and Twiddy, 1992; Ray and Sivakumar, 2009
sapal	vegetable (tuber)	acid	Papua New Guinea	several days	grated, boiled, placed in covered jars	Leuconostoc, LAB, yeasts	Gubag et al., 1996
poi	vegetable (tuber)	acid	Hawaii	several days	steamed, ground, placed in barrels	LAB, Streptococcus	Gubag et al., 1996, Bilger and Young, 1935
tocosh	vegetable (tuber)	acid	South America	months	water immersion	LAB	Werge, 1979; Jimenez et al., 2018
fufu	vegetable (tuber)	acid	West Africa	several weeks	water immersion	LAB	Ray and Sivakumar, 2009; Oyedeji et al., 2013
nattō	vegetable (legume)	alkaline	Japan	1 day	steamed, water immersion	Bacillus subtilis natto	Saono et al., 1986; Murooka et al., 2008
kinema	vegetable (legume)	alkaline	Nepal	1-3 days	soaked, boiled, wrapped	Bacillus, yeasts	Odunfa 1981, Sarkar and Tamang, 1994, Sarkar et al., 1994
idli	vegetable (legume) + grain	acid	India	20 h	ground, mixed with water	Leuconostoc mesenteroides, yeasts	Padhye and Salunkhe 1978;
tempeh	vegetable (legume)	alkaline	Indonesia	1-2 days	soaked, boiled, wrapped	Rhizopus fungi, Bacillus	Steinkraus et al., 1995; Nout and Kiers 2005
soy sauce	vegetable (legume)	alkaline + acid	Asia	1 year	soaked and boiled, then brined	Aspergillus, then LAB and yeasts	Yong and Wood, 1974; Chou and Ling 1999
oncom	vegetable (legume)	alkaline	Indonesia	days to years	soaked, boiled, packed in layers	Rhizopus, Mucor, Neurospora spp. fungi	Sastraatmadja et al., 2002
dawadawa	vegetable (legume)	alkaline	West Africa	days	boiling, pounding, packed in pots	LAB	Dakwa et al., 2005; Deshpande 2000
coffee	vegetable (seed)	acid	Africa	~ 20 hours	pulped and placed in tanks with water	LAB, Bacillus, yeasts and fungi	Avallone et al., 2001
cocoa	vegetable (seed)	acid	Central America	~ 5 days	placed in heaps or depressions with drainage	LAB, Bacillus, yeasts and fungi	Schwan and Wheals, 2014; Schwan and Fleet, 2014
pulque	sap	alcohol	Mexico	12-24h	pulp is placed in closed containers	Zymomas mobilus, LAB, yeasts, proteobacteria	Escalante et al., 2008; Escalante et al, 2011; Valadez-Blanco et al., 2012
mead	honey	alcohol	Africa, Asia, Europe	months to years	diluted, placed in sunlight in open container	yeasts	Iglesias et al., 2014; McGovern et al., 2004; Ramalhosa et al., 2011; Sroka and Tuszynski, 2007; Teramoto et al., 2005
fruit wines	fruit	alcohol	Widespread	days to weeks	placed in sealed containers	yeasts	Battcock et al., 1998
vinegar	fruit or cereals (rice)	acid	Widespread	months	after alcohol fermentation, placed in sealed containers	Acetic acid bacteria, LAB	Solieri and Giudici, 2009; Vegas et al., 2010; Wu et al., 2012
dried sausage	meat	acid	Europe	weeks to months	chopped, placed in casings (some), hung in cool areas	LAB, yeasts, molds	Campbell-Platt, 1995; Danilovic and Savic, 2017; Feiner 2016
country ham	meat	acid	Asia, Europe	several months	salted, dried over time, hot or cold-smoked	LAB, Micrococcus, Staphylococcus, fungi	Bamforth 2008; Campbell-Platt, 1995
satchu	meat	acid	Himalayas	weeks	cut into strips and hung indoors to dry	LAB, Bacillus, Micrococcaceae, yeasts, molds	Rai et al., 2010; Sharma and Sarkar 2015
pemmican	meat	acid	North America	days	dried, chopped, and sealed with fat	*	Campbell-Platt, 1995
twini-digla	internal organs	*	Africa (Sudan)	*	sun- dried, ground, mixed with potash and pressed	*	Campbell-Platt,1995; Dirar 1992
dodery	animal bones	acid	Africa (Sudan)	1-2 weeks	soaked in water, then ground and mixed with potash	Bacillus, Staphylococcus, Micrococcus, LAB, yeasts, proteobacteria	Campbell-Platt,1995; Dirar 1992; Elnabi, 2008
tiroi	shellfish	acid	Oceania	weeks to months	boiled, packed in jars	*	Hudson et al., 2001
kina	shellfish	alkaline	Oceania	weeks to months	buried underground or water immersion	LAB	Hudson et al., 2001
hákarl	shark	alkaline	Iceland	months	packed underground, then hung outdoors	Moraxella, Acinetobacter	Skara et al., 2015; Jonsdottir and Leifsdottir, 2009
ngari	fish	acid	India, Himalayas	months to a year	dried and packed in jars	LAB, Bacillus, Micrococcus, yeasts	Thapa et al., 2004; Jeyaram 2009
surströmming	fish	acid	Europe	months to a year	immersed in brine, packed in barrels	LAB, Haloanaerobium	Skara et al., 2015, Kobayashi et al., 2000
nam-pla	fish	acid	Southeast Asia	18 months	salted and packed in jars	Bacillus, Pseudomonas, Micrococcus, Staphylococcus, Halobacterium	Rattagool et al., 1985; Lopetcharat et al., 2001; Park et al., 2001, McIver et al., 1982
ale	cereal grain	alcohol	Europe	weeeks to months	soaked in water and boiled, then stored in kegs	Yeasts (Saccharomyces spp.)	Lodolo et al., 2008
sake	cereal grain	alcohol	Japan	weeks	soaked, steamed, and left in open containers	Yeast (Saccharomyces) and molds	Uno et al., 2009; Yoshizawa 1999
makgeolli	cereal grain	alcohol	East Asia	1-2 weeks	steamed, stored in containers with inoculated rice starter	Yeast, LAB, fungi	Nile 2015; Kang et al., 2014
chicha	cereal grain	alcohol	Central America	1 week	chewed or malted (soaked,germinated), heated with water	LAB	Cutler and Cardenas, 1947, Elizaquivel et al., 2015
injera	cereal grain	acid	East Africa	several days	ground, mixed with water	LAB, Bacillus, Enterobacteriaceae, yeasts	Fischer et al., 2014; Yetneberk et al., 2004; Stewart and Getachew 1962
kenkey	cereal grain	acid	West Africa	several days	soaked, ground, packed in troughs	LAB, yeasts	Halm et al., 1993
pozol	cereal grain	acid	Central America	hours to days	cooked with lime, ground, wrapped in leaves	LAB, mesophilic bacteria, enterobacteria, molds, yeasts	Wacher et al., 2000; Omar and Ampe, 2000
kumis	dairy	alcohol	Central Asia, S. America	days	loosely covered, occasionally stirred	LAB, yeasts	Kabak and Dobson, 2011; Chaves-Lopez et al., 2011
yogurt	dairy	acid	Middle East	weeks	loosely covered, occasionally stirred	LAB	Innocente et al., 2016; Robinson et al., 2002
cheese	dairy	acid	Europe, Central Asia, N. Africa	days to years	heated, strained, sealed	LAB, mesophilic bacteria, yeasts, molds	Seifu, 2013; Şengül 2006

* Unavailable information

Where possible, descriptions are based on non-commercial/non-industrialized methods

Location origin descriptions are more or less geographically specific depending on available information

Supplementary Table 1. The Ubiquity of Fermentation Technology

Supplementary References

1. Avallone, S., Guyot, B., Brillouet, J. M., Olguin, E., & Guiraud, J. P. (2001). Microbiological and biochemical study of coffee fermentation. Current Microbiology, 42(4), 252–256.
2. Bamforth, C. W. (2008). Food, Fermentation and Micro-organisms. John Wiley & Sons.
3. Başoğlu, F., Şahin, İ., Korukluoğlu, M., Uylaser, V., & Akpinar, A. (1996). A research on the effects of fermentation type and additives on quality and preservation and development of adequate technique in brined vine-leaves production. Turkish Journal of Agriculture and Forestry, 20(6), 535–545.
4. Battcock, M., Azam-Ali, S., & Food and Agriculture Organization of the United Nations. (1998). Fermented Fruits and Vegetables: A Global Perspective. Food & Agriculture Org.
5. Bilger, L. N., Young, H. Y., & Others. (1935). A Chemical Investigation of the Fermentations Occurring in the Process of Poi Manufacture. US Government Printing Office.
6. Campbell-Platt, G. (1994). Fermented foods—a world perspective. Food Research International , 27(3), 253–257.
7. Chaves-López, C., Serio, A., Martuscelli, M., Paparella, A., Osorio-Cadavid, E., & Suzzi, G. (2011). Microbiological characteristics of kumis, a traditional fermented Colombian milk, with particular emphasis on enterococci population. Food Microbiology, 28(5), 1041–1047.
8. Chou, C.-C., & Ling, M.-Y. (1998). Biochemical changes in soy sauce prepared with extruded and traditional raw materials. Food Research International , 31(6), 487–492.
9. Cutler, H. C., & Cardenas, M. (1947). CHICHA, A NATIVE SOUTH AMERICAN BEER. Botanical Museum Leaflets, Harvard University, 13(3), 33–60.
10. Dakwa, S., Sakyi-Dawson, E., Diako, C., Annan, N. T., & Amoa-Awua, W. K. (2005). Effect of boiling and roasting on the fermentation of soybeans into dawadawa (soy-dawadawa). International Journal of Food Microbiology, 104(1), 69–82.
11. Danilović, B., & Savić, D. (2017). Microbial ecology of fermented sausages and dry-cured meats. Fermented Meat Products: Health Aspects, 127–166.
12. Deshpande, S. S. (2000). Fermented Grain Legumes, Seeds and Nuts: A Global Perspective. Food & Agriculture Org.
13. Dirar, H. A. (1992). Sudan's fermented food heritage. Applications of Biotechnology to Traditional Fermented Foods," Ed. by Board on Science and Technology for International Development, National Academy Press, Washington, 27–34.
14. Elizaquível, P., Pérez-Cataluña, A., Yépez, A., Aristimuño, C., Jiménez, E., Cocconcelli, P. S., Vignolo, G., & Aznar, R. (2015). Pyrosequencing vs. culture-dependent approaches to analyze lactic acid bacteria associated to chicha, a traditional maize-based fermented beverage from Northwestern Argentina. International Journal of Food Microbiology, 198, 9–18.
15. Elnabi, A. (2008). Microbiology and Chemical Composition of Fermented Bone Based Food (Dodery) from Darfur-Sudan. Unpublished Dissertation. U of K.

16. Escalante, A., Giles-Gómez, M., Hernández, G., Córdova-Aguilar, M. S., López-Munguía, A., Gosset, G., & Bolívar, F. (2008). Analysis of bacterial community during the fermentation of pulque, a traditional Mexican alcoholic beverage, using a polyphasic approach. International Journal of Food Microbiology, 124(2), 126–134.
17. Escalante, A., Giles-Gómez, M., & Moreno-Terrazas, R. (n.d.). Pulque fermentation. Content.taylorfrancis.com.
18. Feiner, G. (2016). Chapter 7 - Fermented Salami: Non–Heat Treated. In Salami (pp. 111–176). Academic Press.
19. Fischer, M. M., Egli, I. M., Aeberli, I., Hurrell, R. F., & Meile, L. (2014). Phytic acid degrading lactic acid bacteria in tef-injera fermentation. International Journal of Food Microbiology, 190, 54–60.
20. Fleming, H. P., McFeeters, R. F., & Humphries, E. G. (1988). A fermentor for study of sauerkraut fermentation. Biotechnology and Bioengineering, 31(3), 189–197.
21. Gubag, R., Omoloso, D. A., & Owens, J. D. (1996). Sapal: a traditional fermented taro [Colocasia esculenta (L.) Schott] corm and coconut cream mixture from Papua New Guinea. International Journal of Food Microbiology, 28(3), 361–367.
22. Halm, M., Lillie, A., Sørensen, A. K., & Jakobsen, M. (1993). Microbiological and aromatic characteristics of fermented maize doughs for kenkey production in Ghana. International Journal of Food Microbiology, 19(2), 135–143.
23. Hudson, J. A., Hasell, S., Whyte, R., & Monson, S. (2001). Preliminary microbiological investigation of the preparation of two traditional Maori foods (Kina and Tiroi). Journal of Applied Microbiology, 91(5), 814–821.
24. Iglesias, A., Pascoal, A., Choupina, A. B., Carvalho, C. A., Feás, X., & Estevinho, L. M. (2014). Developments in the fermentation process and quality improvement strategies for mead production. Molecules , 19(8), 12577–12590.
25. Innocente, N., Biasutti, M., Rita, F., Brichese, R., Comi, G., & Iacumin, L. (2016). Effect of indigenous Lactobacillus rhamnosus isolated from bovine milk on microbiological characteristics and aromatic profile of traditional yogurt. LWT - Food Science and Technology, 66, 158–164.
26. Jeyaram, K. (2009). Traditional fermented foods of Manipur. Indian Journal of Traditional Knowledge. 8(1),115-121.
27. Jiménez, E., Yépez, A., Pérez-Cataluña, A., Ramos Vásquez, E., Zúñiga Dávila, D., Vignolo, G., & Aznar, R. (2018). Exploring diversity and biotechnological potential of lactic acid bacteria from tocosh - traditional Peruvian fermented potatoes - by high throughput sequencing (HTS) and culturing. LWT - Food Science and Technology, 87, 567–574.
28. Jónsdóttir, G., & Leifsdsóttir, K. (2009). Kennsluefni í matvælafræði ætlað 9.-10. bekk í heimilisfræði. http://skemman.is/handle/1946/3893
29. Kabak, B., & Dobson, A. D. W. (2011). An introduction to the traditional fermented foods and beverages of Turkey. Critical Reviews in Food Science and Nutrition, 51(3), 248–260.
30. Kang, B.-S., Lee, J.-E., & Park, H.-J. (2014). Qualitative and quantitative prediction of volatile compounds from initial amino acid profiles in Korean rice wine (makgeolli) model. Journal of Food Science, 79(6), C1106–C1116.
31. Kobayashi, T., Kimura, B., & Fujii, T. (2000). Strictly anaerobic halophiles isolated from canned Swedish fermented herrings (Surströmming). International Journal of Food Microbiology, 54(1-2), 81–89.
32. Koşar, M., Küpeli, E., Malyer, H., Uylaşer, V., Türkben, C., & Başer, K. H. C. (2007). Effect of Brining on Biological Activity of Leaves of Vitis vinifera L. (Cv. Sultani Çekirdeksiz) from Turkey. Journal of Agricultural and Food Chemistry, 55(11), 4596–4603.
33. Omar, N., & Ampe, F. (2000). Microbial community dynamics during production of the Mexican fermented maize dough pozol. Applied and Environmental Microbiology, 66(9), 3664–3673.
34. Lee, C.-H. (1986). Kimchi; Korean Fermented Vegetable Foods. Journal of the Korean Society of Food Culture, 1(4), 395–402.

35. Lodolo, E. J., Kock, J. L. F., Axcell, B. C., & Brooks, M. (2008). The yeast Saccharomyces cerevisiae– the main character in beer brewing. FEMS Yeast Research, 8(7), 1018–1036.
36. Lopetcharat, K., Choi, Y. J., Park, J. W., & Daeschel, M. A. (2001). FISH SAUCE PRODUCTS AND MANUFACTURING: A REVIEW. Food Reviews International, 17(1), 65–88.
37. Lv, H.-P., Zhang, Y.-J., Lin, Z., & Liang, Y.-R. (2013). Processing and chemical constituents of Pu-erh tea: A review. Food Research International , 53(2), 608–618.
38. McGovern, P. E., Zhang, J., Tang, J., Zhang, Z., Hall, G. R., Moreau, R. A., Nuñez, A., Butrym, E. D., Richards, M. P., Wang, C.-S., Cheng, G., Zhao, Z., & Wang, C. (2004). Fermented beverages of pre- and proto-historic China. Proceedings of the National Academy of Sciences of the United States of America, 101(51), 17593–17598.
39. McIver, R. C., Brooks, R. I., & Reineccius, G. A. (1982). Flavor of fermented fish sauce. Journal of Agricultural and Food Chemistry, 30(6), 1017–1020.
40. Murooka, Y., & Yamshita, M. (2008). Traditional healthful fermented products of Japan. Journal of Industrial Microbiology & Biotechnology, 35(8), 791–798.
41. Nile, S. H. (2015). The nutritional, biochemical and health effects of makgeolli--a traditional Korean fermented cereal beverage. Journal of the Institute of Brewing. Institute of Brewing , 121(4), 457–463.
42. Nout, M. J. R., & Kiers, J. L. (2005). Tempe fermentation, innovation and functionality: update into the third millenium. Journal of Applied Microbiology, 98(4), 789–805.
43. Odunfa, S. A. (1981). Microorganisms associated with fermentation of African locust bean (Parkia filicoidea) during iru preparation. Journal of Plant Foods, 3(4), 245–250.
44. Oyedeji, O., Ogunbanwo, S. T., & Onilude, A. A. (2013). Predominant lactic acid bacteria involved in the traditional fermentation of fufu and ogi, two Nigerian fermented food products. Food and Nutrition Sciences , 4(11), 40.
45. Padhye, V. W., & Salunkhe, D. K. (1978). Biochemical Studies on black gram (Phaseolus mung L.) 111. Fermentation of the black gram and rice blend and its influence on the in vitro digestibility of the proteins. Journal of Food Biochemistry, 2(4), 327–347.
46. Park, J.-N., Fukumoto, Y., Fujita, E., Tanaka, T., Washio, T., Otsuka, S., Shimizu, T., Watanabe, K., & Abe, H. (2001). Chemical Composition of Fish Sauces Produced in Southeast and East Asian Countries. Journal of Food Composition and Analysis: An Official Publication of the United Nations University, International Network of Food Data Systems, 14(2), 113–125.
47. Rai, A. K., Tamang, J. P., & Palni, U. (2010). Microbiological studies of ethnic meat products of the Eastern Himalayas. Meat Science, 85(3), 560–567.
48. Ramalhosa, E., Gomes, T., Pereira, A. P., Dias, T., & Estevinho, L. M. (2011). Mead production: tradition versus modernity. Advances in Food and Nutrition Research, 63, 101–118.
49. Rattagool, P. (1985). Fermented fish products of South East Asia. Trop Sci, 25, 61–73.
50. Ray, R. C., & Sivakumar, P. S. (2009). Traditional and novel fermented foods and beverages from tropical root and tuber crops. International Journal of Food Science & Technology, 44(6), 1073–1087.
51. Robinson, R. K., Tamime, A. Y., & Wszolek, M. (2002). Microbiology of fermented milks. Dairy Microbiology Handbook: The Microbiology of Milk and Milk Products, 468.

52. Saono, S., Hull, R. R. & Dhamcharee, B. (1986). Concise handbook of indigenous fermented foods in the ASCA countries. Indonesian Institute of Sciences.
53. Sarkar, P. K., & Tamang, J. P. (1994). The influence of process variables and inoculum composition on the sensory quality of kinema. Food Microbiology, 11(4), 317–325.
54. Sarkar, P., Tamang, J. P., Cook, P. E., & Owens, J. (1994). Kinema—a traditional soybean fermented food: proximate composition and microflora. Food Microbiology, 11(1), 47–55.
55. Sastraatmadja, D. D., Tomita, F., & Kasai, T. (2002). Production of High-Quality Oncom, a Traditional Indonesian Fermented Food, by the Inoculation with Selected Mold Strains in the Form of Pure Culture and Solid Inoculum. Journal of the Graduate School of Agriculture, Hokkaido University= 北海道大学大学院農学研究科紀要, 70(2), 111–127.
56. Schwan, R. F., & Fleet, G. H. (2014). Cocoa and Coffee Fermentations. CRC Press.
57. Schwan, R. F., & Wheals, A. E. (2004). The microbiology of cocoa fermentation and its role in chocolate quality. Critical Reviews in Food Science and Nutrition, 44(4), 205–221.
58. Seifu, E. (2013). Chemical composition and microbiological quality of Metata Ayib: a traditional Ethiopian fermented cottage cheese. Food and Chemical Toxicology: An International Journal Published for the British Industrial Biological Research Association, 20(1), 93.
59. Şengül, M. (2006). Microbiological characterization of Civil cheese, a traditional Turkish cheese: microbiological quality, isolation and identification of its indigenous Lactobacilli. World Journal of Microbiology & Biotechnology, 22(6), 613–618.
60. Sharma, A., & Sarkar, P. K. (2015). Microbial diversity in Ethno-fermented foods of Indian Himalayan Region. ENVIS Bulletin Himalayan Ecology, 23, 85.
61. Skåra, T., Axelsson, L., Stefánsson, G., Ekstrand, B., & Hagen, H. (2015). Fermented and ripened fish products in the northern European countries. Journal of Ethnic Foods, 2(1), 18–24.
62. Solieri, L., & Giudici, P. (2009). Vinegars of the World. In L. Solieri & P. Giudici (Eds.), Vinegars of the World (pp. 1–16). Springer Milan.
63. Sroka, P., & Tuszyński, T. (2007). Changes in organic acid contents during mead wort fermentation. Food Chemistry, 104(3), 1250–1257.
64. Stamer, J. R., Stoyla, B. O., & Dunckel, B. A. (1971). Growth rates and fermentation patterns of lactic acid bacteria associated with the sauerkraut fermentation. Journal of Milk and Food Technology, 34(11), 521–525.
65. Steinkraus, K. H. (1994). Nutritional significance of fermented foods. Food Research International , 27(3), 259–267.
66. Stewart, R. B., & Getachew, A. (1962). Investigations of the nature of Injera. Economic Botany, 16(2), 127–130.
67. Tamang, J. P., & Sarkar, P. K. (1993). Sinki: a traditional lactic acid fermented radish tap root product. The Journal of General and Applied Microbiology, 39(4), 395–408.
68. Teramoto, Y., Sato, R., & Ueda, S. (2005). Characteristics of fermentation yeast isolated from traditional Ethiopian honey wine, ogol. African Journal of Biotechnology, 4(2), 160–163.
69. Thapa, N., Pal, J., & Tamang, J. P. (2004). Microbial Diversity in Ngari, Hentak and Tungtap, Fermented Fish Products of North-East India. World Journal of Microbiology & Biotechnology, 20(6), 599.
70. Uno, T., Itoh, A., Miyamoto, T., Kubo, M., Kanamaru, K., Yamagata, H., Yasufuku, Y., & Imaishi, H. (2009). Ferulic Acid Production in the Brewing of Rice Wine (Sake). Journal of the Institute of Brewing. Institute of Brewing , 115(2), 116–121.

71. Valadez-Blanco, R., Bravo-Villa, G., Santos-Sánchez, N. F., Velasco-Almendarez, S. I., & Montville, T. J. (2012). The Artisanal Production of Pulque, a Traditional Beverage of the Mexican Highlands. Probiotics and Antimicrobial Proteins, 4(2), 140–144.
72. Vegas, C., Mateo, E., González, A., Jara, C., Guillamón, J. M., Poblet, M., Torija, M. J., & Mas, A. (2010). Population dynamics of acetic acid bacteria during traditional wine vinegar production. International Journal of Food Microbiology, 138(1-2), 130–136.
73. Wacher, C., Cañas, A., Bárzana, E., Lappe, P., Ulloa, M., & Owens, J. D. (2000). Microbiology of Indian and Mestizo pozol fermentations. Food Microbiology, 17(3), 251–256.
74. Wang, Q., Peng, C., & Gong, J. (2011). Effects of enzymatic action on the formation of theabrownin during solid state fermentation of Pu-erh tea. Journal of the Science of Food and Agriculture. https://onlinelibrary.wiley.com/doi/abs/10.1002/jsfa.4480
75. Werge, R. W. (1979). Potato Processing In The Central Highlands Of Peru Vol-7. MPKV; Maharastra. http://14.139.56.90/bitstream/1/2056030/1/MPKV-654.pdf
76. Westby, A., & Twiddy, D. R. (1992). Characterization ofgari andfu-fu preparation procedures in Nigeria. World Journal of Microbiology & Biotechnology, 8(2), 175–182.
77. Wu, J. J., Ma, Y. K., Zhang, F. F., & Chen, F. S. (2012). Biodiversity of yeasts, lactic acid bacteria and acetic acid bacteria in the fermentation of "Shanxi aged vinegar", a traditional Chinese vinegar. Food Microbiology, 30(1), 289–297.
78. Yong, F. M., & Wood, B. J. B. (1974). Microbiology and Biochemistry of Soy Sauce Fermentation. In D. Perlman (Ed.), Advances in Applied Microbiology (Vol. 17, pp. 157–194). Academic Press.
79. Yoshizawa, K. (1999). Sake: Production and flavor. Food Reviews International, 15(1), 83–107.
